# Diagnosis and Management of Atypical Chronic Myeloid Leukemia with a t(2;13)(q33;q12) Translocation

**DOI:** 10.1155/2022/4628183

**Published:** 2022-05-04

**Authors:** John S. Wang, Omar Elghawy, Brett R. Kurpiel, Michael G. Douvas

**Affiliations:** School of Medicine, University of Virginia, Charlottesville, Virginia, USA

## Abstract

Atypical chronic myeloid leukemia (aCML) is a rare myeloproliferative disorder that shares clinical features with chronic myeloid leukemia but lacks the classic t(9;22) *BCR-ABL1* translocation and features prominent dysgranulopoiesis and granulocytic dysplasia. Challenges of this diagnosis include clinical and biologic heterogeneity, the high risk of transformation to acute myeloid leukemia, and the lack of standard treatment options. Allogeneic hematopoietic stem cell transplant is likely the preferred treatment, but this can be limited by patient psychosocial support, age, concomitant medical conditions, and availability of an appropriate donor. We report the case of a 61-year-old male with no significant past medical history diagnosed with aCML with a rare t(2;13)(q33;q12). He presented with weight loss, night sweats, splenomegaly, hyperleukocytosis, a leukoerythroblastic differential with a predominant neutrophilia, anemia, and thrombocytopenia. Subsequent peripheral blood and bone marrow studies lead to the diagnosis of aCML. He was recommended to undergo an allogeneic stem cell transplant evaluation and declined. He was initially treated with hydroxyurea and imatinib to which he responded for approximately three years. After clinical progression, he was treated with sorafenib, a multiprotein kinase inhibitor more commonly used in the treatment of hepatocellular and renal cell carcinoma due to its off target FLT3 inhibition. The patient achieved complete hematologic response which has been sustained for 7 years with tolerable side effects.

## 1. Introduction

Atypical chronic myeloid leukemia (aCML) is a myeloproliferative neoplasm with a poor prognosis due to its clinical and biologic heterogeneity, its high risk of transformation to acute myeloid leukemia, and its lack of a standardized treatment protocol [1]. aCML is characterized by marked leukocytosis/granulocytosis but unlike classic CML, shows no evidence of the classic t(9;22) *BCR-ABL1* translocation by cytogenetic studies, polymerase chain reaction (PCR), or fluorescence situ hybridization (FISH) [1, 2]. The morphologic features of aCML share similarities with CML and include leukocytosis, granulocytosis, and a spectrum of immature granulocytes in peripheral blood. aCML, however, is unique in that patients also exhibit features of prominent dysgranulopoiesis and granulocytic dysplasia. Patients often present with signs and symptoms of anemia and hepatosplenomegaly on physical exam. The disease is extremely rare with an estimated incidence of less than one in a million people per year [3]. In this case report, we present an older man presenting with aCML with a t(2;13)(q33;q12) who attained a complete clinical and hematologic remission when treated with the protein kinase inhibitor sorafenib after previously having a partial response to imatinib.

## 2. Case Report

**Figure 1:**
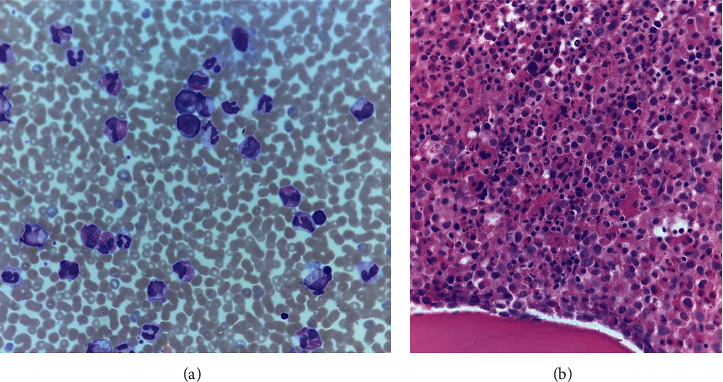
(a) Peripheral blood smear demonstrating a leukocytosis with myeloid left shift, basophilia, eosinophilia, polycythemia, and thrombocytopenia; Wright-Giemsa, original image ×1,000, oil immersion. (b) High-power image of the bone marrow biopsy highlighting the dramatic myeloid predominance with only few, rare erythroid precursors and small, hypolobated megakaryocytes; hematoxylin-eosin, original image ×500, oil immersion.

**Figure 2:**
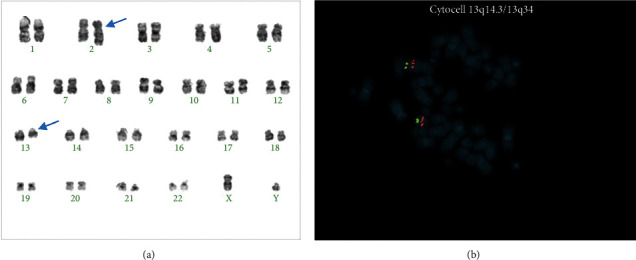
(a) Karyotype images showing t(2;13). (b) FISH image demonstrating the probes for chromosome 13 hybridizing to the normal chromosome 13 (small chromosome) and chromosome 2 (large chromosome), demonstrating the translocation of chromosome 13 material to chromosome 2.

**Figure 3:**
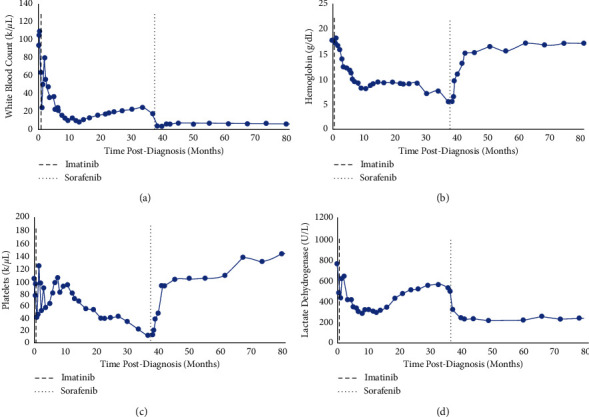
(a) WBC count. (b) Hemoglobin. (c) Platelets. (d) LDH for the patient over time from date of presentation to current. The thick dashed line represents the time of imatinib administration while the thin dashed line represents the time of therapy conversion to sorafenib.

**Figure 4:**
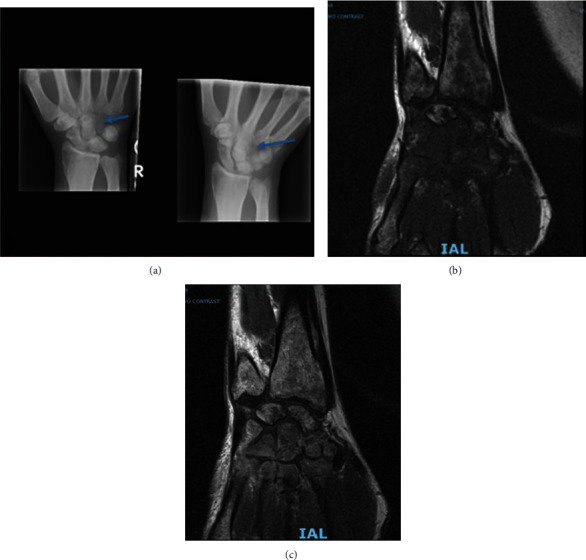
(a) Plain film X-ray of the right hand reveals diffuse severe lucency of the entire hamate bone (blue arrows) with a thin peripheral cortex visible. (b) T1 MRI. (c) T2-weighted MRI with contrast of the right hand reveals severe bone marrow replacement throughout the distal radius and ulna, carpal bones, and metacarpals with complete replacement of the trapezoid, capitate, and hamate.

An otherwise healthy 61-year-old male presented for evaluation of weight loss, night sweats, and left upper quadrant discomfort. He had no significant medical history, and his family history was significant only for hypothyroidism. Physical exam revealed a well appearing man with palpable splenomegaly to 5 fingerbreadths below the left costal margin in the midclavicular line. A complete blood count with differential revealed mild thrombocytopenia (103 K), borderline elevated hemoglobin (17.4), and leukocytosis to 93 k with a leukoerythroblastic differential (PMN 44%, bands 8%, myelocytes/metamyelocytes 14%, promyelocytes 8%, blasts 2%, eosinophils 17%, and basophils 2%). Review of his peripheral blood smear showed significant dysgranulopoiesis with a leukoerythroblastic differential felt to be most consistent with aCML (Figure 1(a)). Routine laboratory workup revealed no significant abnormalities, except a moderately elevated lactate dehydrogenase of 755 (ULN 250) and a mildly elevated uric acid of 8.8 (ULN 7.2). A bone marrow aspiration was performed and showed findings similar to peripheral blood with 57% neutrophils and bands, 17% eosinophils, and no elevation in blasts. The bone marrow core biopsy showed marked hypercellularity of 100%, notable granulocytic dysplasia, and myeloid hyperplasia with a markedly elevated myeloid: erythroid ratio of 30 : 1 (Figure 1(b)). Cytogenetic analysis was negative for *BCR-ABL1* translocation by FISH but revealed a balanced reciprocal translocation between chromosomes 2 and 13 t(2;13)(q33;q12) in all 19 cells analyzed by karyotype (Figure 2(a)). FISH analysis for translocations involving *PDGFRA* and *PDGFRB* was negative as was a quantitative PCR for p210 *BCR-ABL1*. FISH using the CytoCell 13q14.3/13q34 break-apart probe for 13q showed no evidence of disruption but did demonstrate that this probe had been translocated to chromosome 2 (Figure 2(b)). The diagnosis of aCML was made in concordance with the WHO diagnostic criteria for aCML [4]. Due to the lack of a standard medical regimen for a diagnosis of aCML with a t(2;13) translocation, a stem cell transplant evaluation was recommended, but ultimately declined by the patient. He was started initially on both hydroxyurea and in the process of evaluation on imatinib, given the strikingly similar presentation to CML and lack of other available treatment modalities. Hydroxyurea was stopped after 5 months after significant improvement in leukocytosis. Somewhat unexpectedly, the patient continued to clinically respond to imatinib for approximately three years with normalization of white blood cell count and decrease of LDH (Figure 3), regression of splenomegaly, and overall increased quality of life. After three years, however, the patient developed worsening thrombocytopenia and anemia despite subsequent dose reductions of imatinib. He also developed severe right-hand pain that was unresponsive to NSAIDs and prednisone. A plain film of the hand revealed diffuse severe lucency of the entire hamate bone with a thin peripheral cortex visible, and a subsequent MRI revealed severe bone marrow replacement throughout the distal radius and ulna, carpal bones, and metacarpals with complete abnormal marrow replacement of the trapezoid, capitate, and hamate (Figure 4). There was a strong concern for possible transformation to acute myeloid leukemia (AML), but the patient did not have peripheral blasts on the routine blood smear. The patient declined a repeat bone marrow biopsy, next-generation sequencing, and recommended transfusions for platelets of 11 K/*μ*L and Hgb of 5.3 g/dL. Given a previous case report of a patient with a similar presentation and a t(2;13)(q33,q12) involving the *FLT3* locus, a search of tyrosine kinase inhibitors with activity on the FLT3 protein ensued [5]. Due to the patient's worsening blood counts and a lack of alternative treatments options, the patient was started on sorafenib therapy as it was the only commercially available FLT3 inhibitor at the time. The patient had rapid clinical improvement of both blood counts and pain and swelling of his right hand after initiation of sorafenib. He ultimately normalized his WBC count and differential, hemoglobin, and LDH with a resolution of splenomegaly on exam, normalization of his hand exam, resolution of bone pain, and improvement in his platelet count to mild stable thrombocytopenia of approximately 100 K/*μ*L (Figure 3). He has now been on sorafenib for over seven years with mild and manageable side effects of peripheral edema, hypertension requiring single-agent antihypertensive medication, and intermittent loose stools.

## 3. Discussion

Our patient presented with atypical CML defined by his *BCR-ABL1*(-) status in the setting of marked leukocytosis/granulocytosis and prominent dysgranulopoiesis, and granulocytic dysplasia. This is the first case reported in the literature of a patient with myeloproliferative neoplasm with t(2;13)(q33;q12) being successfully treated with sorafenib. This case complements the atypical CML case described by Grand et al. [6]. Their patient had a similar presentation with elevated white blood cells, anemia, thrombocytopenia, and splenomegaly [6]. They confirmed via molecular testing that their patient with a t(2;13)(p13;q12) translocation had a fusion of *SPTBN1-FLT3* with fluorescent in-situ hybridization and PCR confirming the fusion of the two genes. The *SPTBN1* gene encodes a nonerythrocytic beta-spectrin that is a tetrameric cytoskeletal protein with characteristic two coiled-coil domains that are preserved when fused with the entire tyrosine kinase domain of FLT3. The chimeric fusion was described to result in a constitutively active *FLT3* mutant that phosphorylates *STAT5* protein [7–9] which has been implicated in upregulating proliferative pathways in the myeloid lineage, particularly in AML patients [10, 11]. While the patient in Grant et al. was treated with an allogeneic stem cell transplant and with donor lymphocyte infusion after cytogenetic relapse years later, patient-derived cell growth was shown to be inhibited in vitro by *FLT3* inhibitors in a dose-dependent manner [6]. We posit that a similar pathway via a chimeric *FLT3* mutant is responsible for the atypical CML observed in our patient and his subsequent response to tyrosine kinase inhibitor (TKI) therapy [12]. Unfortunately, molecular studies were not performed on our patient at the time of his presentation or at time of progression due in part to the patient's wishes.

Conventional treatment for classical CML with the t(9;22) *BCR-ABL* translocation is therapy with tyrosine kinase inhibitors such as imatinib which are very effective [13, 14]. Interestingly, in our case, we observed the patient to respond initially to imatinib therapy in the absence of the t(9;22) translocation for nearly three years. There are two potential explanations for our patient's partial response to imatinib. One possibility is that our patient developed a myeloid or lymphoid neoplasm with eosinophilia and rearrangement of the PDGFRA, PDGFRB, FGFR1, or PCM1-JAK2 genes leading to a partial response to imatinib [15]. Assessment with FISH for the most common PDGFRA/B gene translocations in our patient was negative, and while the presence of a cryptic rearrangement could explain our patient's partial response to imatinib, it would not explain his full response to sorafenib. More likely, we speculate that imatinib had some efficacy, albeit temporary and less complete, in inhibiting the t(2;13)(q33;q12) chimeric *FLT3* kinase in our case.

In light of our patient's clinical course, it is noted to be important to both report rare clinical presentations for the potential benefit of other similar patients and to attempt to perform appropriate molecular testing whenever possible to advance understanding of the pathophysiology of rare diseases. Sorafenib, a drug most often used in the treatment of hepatocellular and renal cell carcinoma, would not have been chosen as the treatment modality for this patient if not for the previous case report detailing in vitro response to various TKIs [16–18]. As demonstrated in the case presented here, the recognition of the t(2;13)(q33;q12) translocation and the prior case report were the keys to the successful treatment for our patient.

## Data Availability

All data generated or analyzed during this study are included in this article. Further enquiries can be directed to the corresponding author.

## Additional Points

(1) Atypical CML is a rare variant of CML that shares many clinical characteristics of CML but lacks the *BCR-ABL1* t(9;22) of CML and features prominent dysgranulopoiesis and granulocytic dysplasia. (2) Atypical CML involving the (q33;q12) locus (that includes *FLT3*) may respond well to sorafenib or other more potent FLT3 inhibitors now commercially available.
